# Correction of Hyponatremia May Be a Treatment Stratification Biomarker: A Two-Stage Systematic Review and Meta-Analysis

**DOI:** 10.3390/jcm7090262

**Published:** 2018-09-07

**Authors:** Francisco Herrera-Gómez, Diana Monge-Donaire, Carlos Ochoa-Sangrador, Juan Bustamante-Munguira, Eric Alamartine, F. Javier Álvarez

**Affiliations:** 1 Pharmacology and Therapeutics, Faculty of Medicine, University of Valladolid, Avenida Ramón y Cajal, 7, 47005 Valladolid, Spain; alvarez@med.uva.es; 2 Nephrology, Hospital Virgen de la Concha—Sanidad de Castilla y León, 49022 Zamora, Spain; 3 Intensive Care Medicine, Hospital Virgen de la Concha—Sanidad de Castilla y León, 49022 Zamora, Spain; dianadonaire@gmail.com; 4 Research Unit, Hospital Virgen de la Concha—Sanidad de Castilla y León, 49022 Zamora, Spain; cochoas2@gmail.com; 5 Cardiac Surgery, Hospital Clínico Universitario de Valladolid—Sanidad de Castilla y León, 47003 Valladolid, Spain; jbustamantemunguira@gmail.com; 6 Nephrology, Dialysis and Transplantation, Centre Hospitalier Universitaire de Saint-Etienne, 42270 Saint-Priest-en-Jarez, France; eric.alamartine@univ-st-etienne.fr; 7 CEIm Área de Salud Valladolid Este, Hospital Clínico Universitario de Valladolid—Sanidad de Castilla y León, 47003 Valladolid, Spain

**Keywords:** hyponatremia, biomarkers, drug evaluation, heart failure, ascites

## Abstract

Changes in serum sodium concentration ([Na^+^]_serum_) can permit evaluation of the treatment effect of vasopressin antagonists (vaptans) in patients with worsening heart failure (HF) or cirrhotic ascites; that is, they may act as a treatment stratification biomarker. A two-stage systematic review and meta-analysis were carried out and contextualized by experts in fluid resuscitation and translational pharmacology (registration ID in the International Prospective Register of Systematic Reviews (PROSPERO): CRD42017051440). Meta-analysis of aggregated dichotomous outcomes was performed. Pooled estimates for correction of hyponatremia (normalization or an increase in [Na^+^]_serum_ of at least 3–5 mEq/L) under treatment with vaptans (Stage 1) and for clinical outcomes in both worsening HF (rehospitalization and/or death) and cirrhotic ascites (ascites worsening) when correction of hyponatremia is achieved (Stage 2) were calculated. The body of evidence was assessed. Correction of hyponatremia was achieved under vaptans (odds ratio (OR)/95% confidence interval (95% CI)/I^2^/number of studies (n): 7.48/4.95–11.30/58%/15). Clinical outcomes in both worsening HF and cirrhotic ascites improved when correction of hyponatremia was achieved (OR/95% CI/I^2^/n: 0.51/0.26–0.99/52%/3). Despite the appropriateness of the study design, however, there are too few trials to consider that correction of hyponatremia is a treatment stratification biomarker. Patients with worsening HF or with cirrhotic ascites needing treatment with vaptans, have better clinical outcomes when correction of hyponatremia is achieved. However, the evidence base needs to be enlarged to propose formally correction of hyponatremia as a new treatment stratification biomarker. Markers for use with drugs are needed to improve outcomes related to the use of medicines.

## 1. Introduction

Hyponatremia is defined as a serum sodium concentration ([Na^+^]_serum_) of less than 135 mEq/L [1]. It is the most common electrolyte abnormality in clinical practice [2]. The understanding of its multifactorial etiology, pathophysiology and clinical presentation are essential in managing patients [3,4].

In the hospital, hypervolemic hyponatremia can be encountered [1], and it appears when water retention exceeds that of sodium. Decompensated heart failure (HF) and cirrhotic ascites episodes are two important causes [2]. Most remarkably, HF and cirrhosis constitute frequent causes of hospitalization [5], and in such patients, hyponatremia is considered as a poor prognostic factor [5,6].

Nevertheless, in both HF and liver cirrhosis patients, hyponatremia reflects a higher activity of arginine vasopressin (AVP), inducing electrolyte-free water retention by binding V2 receptors [7,8]. Vaptans, nonpeptide vasopressin-receptor antagonists, increase electrolyte free-water excretion (aquaresis) and, consequently, serum osmolality [9]. Considering that normalization of [Na^+^]_serum_ is pivotal in HF and advanced cirrhosis, vaptans offer a new treatment approach for these diseases sharing a maladaptive AVP response with the syndrome of inappropriate antidiuretic hormone secretion (SIADH).

Recently, there have been higher expectations that biological processes can be measured and utilized in order to influence clinical decision-making. Nevertheless, several hard issues preventing drug evaluation using biomarkers need to be solved [10]. With the aim to elucidate whether correction of hyponatremia is a treatment stratification biomarker [11], a two-stage systematic review and meta-analysis were performed to summarize the efficacy of the V_1A_/V_2_-receptor antagonist conivaptan [12,13,14,15] and the V_2_-receptor antagonists lixivaptan [16,17,18,19,20], satavaptan [21,22,23] and tolvaptan [24,25,26,27,28,29,30,31,32,33,34,35,36] in relation to changes in [Na^+^]_serum_ in patients with worsening HF or cirrhotic ascites.

## 2. Materials and Methods

A systematic mapping (Stage 1) followed by an in-depth systematic review (Stage 2) were conducted and reported according to the Preferred Reporting Items for Systematic Reviews and Meta-Analyses (PRISMA) guidelines [37]. A systematic review protocol was developed and registered in the International Prospective Register of Systematic Reviews (PROSPERO) under the following registration ID: CRD42017051440 (final version and revision history of this protocol are available at: http://www.crd.york.ac.uk/PROSPERO/display_record.php?ID=CRD42017051440). A multidisciplinary supervision mechanism provided by an expert advisory group that brought together experts in fluid resuscitation (EA and JB-M) and translational pharmacology (FH-G and FJA) was planned for contextualizing the search findings [38].

Table 1 presents the review question at each of the two planned systematic review stages and the participants, intervention(s)/exposure(s) and comparators being studied. At both stages, randomized controlled trials (RCT), an extension of follow-up of such trials and post-hoc or subgroup analysis of RCTs were requested. The primary outcome was the clinical effect of vaptans in patients with worsening HF and cirrhotic ascites assessed by changes in [Na^+^]_serum_. Secondary outcomes were the correction of hyponatremia (defined as normalization or increase in [Na^+^]_serum_ of 3–5 mEq/L or more) and response to therapy with vaptans.

MEDLINE via PubMed, Ovid MEDLINE^®^ and Web of Science, EMBASE via Elsevier’s Scopus and The Cochrane Central Register of Controlled Trials (CENTRAL) were searched through October 2017. Database-specific search strategies were developed using terms related to the type of study to be included for both stages, terms related to eligible participants and the intervention for Stage 1 and terms related to medical conditions motivating this study for Stage 2. Searches in electronic databases were supplemented by searching ClinicalTrials.gov and grey literature sources. The DART-Europe E-Theses portal and Open Access Theses and Dissertations (OATD) were interrogated to identify relevant PhD and Master’s theses. Manual searches in meeting abstract archives of the Heart Failure Society of America (HFSA) Annual Scientific Meeting 2003–2017, the European Society of Cardiology (ESC) Heart Failure congress 2001–2016, the European Association for the Study of the Liver (EASL) The International Liver Congress 2004–2017 and the American Association for the Study of Liver Diseases (AASLD) The Liver Meeting 2001–2017 were conducted to retrieve relevant abstracts. Finally, to ensure literature saturation, a cited reference search of all eligible publications was carried out using Web of Science to identify all studies citing the included studies. The full search strategy is available online at: https://www.crd.york.ac.uk/PROSPEROFILES/51440_STRATEGY_20171218.pdf.

Screening of titles/abstracts and, subsequently, full text report examination of potentially eligible articles were carried out independently and in duplicate by two different reviewer teams formed by CO-S and DM-D for Stage 1 and by DM-D and FH-G for Stage 2. Disagreements were resolved by discussion or referral to a third author (FJA). Corresponding authors of the included studies were contacted whenever possible to retrieve missing information and to confirm study details.

Anonymized datasets corresponding to each of the two stages, describing the characteristics of studies and their participants, interventions, comparators and outcomes recorded in trials eligible, were constructed. Before any analysis, risk of bias was assessed using the standard tool developed by the Cochrane Collaboration [39]. A two-stage meta-analysis of aggregate-level data was planned (CO-S, DM-D and FH-G). The overall odds ratio (OR) and its 95% confidence interval (95% CI) for the outcome of correction of hyponatremia under treatment with vaptans (Stage 1) and for clinical outcomes in both worsening HF (rehospitalization and/or death) and cirrhotic ascites (ascites worsening, defined by either the need for therapeutic paracentesis or an increase in diuretic dosage or weight gain of at least 2 kg) when correction of hyponatremia was achieved (Stage 2), were obtained (Mantel–Haenszel random-effect model meta-analysis). Examination of heterogeneity (I² and χ²) and the presence of reporting bias (visual inspection of funnel plots of the estimates against their standard errors) was performed. At Stage 2, calculation of the regression coefficient corresponding to vaptans and the treatment objective of correction of hyponatremia (potential effect modifiers) was attempted (random-effects meta-regression). Review Manager (RevMan) software Version 5.3 (The Cochrane Collaboration, London, UK) was used for meta-analysis, and the ‘metareg’ macro from Stata Version 12.1 (StataCorp, College Station, TX, USA) was dedicated to meta-regression. A two-staged systematic narrative synthesis with non-quantitative data was also presented [40].

Codependency when combining technologies related to the treatment and the potential biomarker was assessed using an adaptation of Merlin’s tool included in the guidelines for preparing a submission to the Pharmaceutical Benefits Advisory Committee (PBAC) from the Department of Health of Australia (CO-S and FH-G) [41,42]. The tool sections of economic evaluation and use of the medicine in practice were not considered.

## 3. Results

A total of 2075 unique citations were identified throughout the Stage 1 search process. Of these, 25 fulfilled the eligibility criteria [12,13,14,15,16,17,18,19,20,21,22,23,24,25,26,27,28,29,30,31,32,33,34,35,36]. Nearly all reports were peer-reviewed journal articles that, in association with two meeting abstracts [19,34], presented the results from 15 RCTs. Out of these trials, the three following were considered eligible for the in-depth systematic review: Satavaptan dose-ranging study in Hyponatremic patients with Cirrhotic AsciTes (HypoCAT) [23], the Acute and Chronic Therapeutic Impact of a Vasopressin antagonist in Congestive Heart Failure (ACTIV in CHF) study [26,27], and the Efficacy of Vasopressin Antagonism in Heart Failure Outcome Study with Tolvaptan (EVEREST) [28,29,30]. No new reports were identified at the in-depth systematic review search process. At both stages, irrelevant citations were mostly observational studies and opinion narrative reviews. Figure 1 presents the PRISMA flowcharts corresponding to both systematic review stages and the search results obtained [43]. Table 2 shows all eligible studies.

Overall, the trials were of moderate quality (Supplementary Table S1). Data from 2238 participants were analyzed. Correction of hyponatremia was achieved after 2–5 days of treatment with vaptans (59.27%) and, later, after placebo (18.91%). Figure 2 shows the calculation of the pooled OR for this treatment objective, which was 7.48 with 95% CI 4.95–11.30 (*p* < 0.00001, I^2^ = 58%) [12,13,14,15,16,17,18,19,20,21,22,23,24,25,26,27,28,29,30,31,32,33,34]. Asymmetry in the funnel plot was noted [44].

Rehospitalization and/or death among patients with worsening HF and ascites worsening among liver cirrhosis patients presenting ascites were less frequent in those having achieved correction of hyponatremia, mostly under treatment with vaptans (tolvaptan and satavaptan). The pooled OR was 0.51 with 95% CI 0.26–0.99 (*p* = 0.05, I^2^ = 52%, Figure 3) [23,26,27,28,29,30]. Meta-regression with tolvaptan and satavaptan, and with correction of hyponatremia, was not possible given the number of studies included [45]. Outcomes were evaluated until 30–60 days of treatment. Only qualitatively, efficacy outcomes (changes in body weight, edema and other endpoints related to electrolyte free-water excretion) improved under vaptans.

Although it was not clearly specified, the three trials included at Stage 2 used a retrospective biomarker-stratified design, providing low-level direct evidence on codependent health technologies, leading to a benefit from vaptans for patients with worsening HF and cirrhotic ascites (Table 3) [41,42,46].

## 4. Discussion

Clinical outcomes in patients with worsening HF or with cirrhotic ascites improved when correction of hyponatremia (normalization or an increase in [Na^+^]_serum_ of at least 3–5 mEq/L) was achieved [23,26,27,28,29,30]. There is a little but adequate evidentiary support that seeks to relate changes in [Na^+^]_serum_ to treatment with vaptans, leading to the suggestion that correction of hyponatremia may be a new predictive or treatment stratification biomarker.

To date, vaptans have demonstrated an effect on [Na^+^]_serum_ [47,48], but no systematic review and meta-analysis has confirmed the clinical impact of this effect in both worsening HF [49,50,51,52,53] and cirrhotic ascites [54,55,56]. Importantly, although these drugs are used after water restriction and diuretics, only some molecules have been studied clinically, and only two vaptans are actually used in the clinic. Therefore, available evidence is limited. In addition, the findings of this study could be interpreted as favoring tolvaptan. However, the summary presented here constitutes an evaluation of the effect of vasopressin-receptor antagonists as a pharmacologic group, and not an analysis of the benefits from only one drug.

Improvement in [Na^+^]_serum_ can aid the identification of good and poor responders to vaptans. Most remarkably, the evidentiary process of linking biological processes and clinical outcomes under the effect of drugs such that it can be adopted into clinical practice, also called biomarker qualification, is very challenging [10]. In this context, identifying the proper character of the biomarker and whether it is useful for predicting variations in the clinical effect of a medicine or treatment combining medicines (causal relationship) must be known [41,42,46]. Eligible trials at Stage 2 used a retrospective biomarker-stratified design; that is, trials randomized eligible participants to vaptans or placebo and measured the effect of treatment in terms of health outcomes (clinical improvement of decompensated HF or cirrhotic ascites) across patient subgroups defined by the biomarker status (correction of hyponatremia) [41,42]. This trial design provides direct evidence, albeit of a lower level, to suggest that correction of hyponatremia may predict the clinical effect of vaptans. However, meta-regression was not possible, so the evidence base needs to be enlarged to propose this treatment objective as a new treatment stratification biomarker for patients.

Recently and at a rapid pace, several biomarkers are appearing [10]. The main purpose is to enable more efficient decision-making by clinicians when facing the patient. From the perspective of health technology assessment (HTA), new markers for use with drugs must be an aid for ascertaining the best medicine for a given disease, as well as the most appropriate doses of this medicine; that is, for personalizing therapies [57]. Transformation of the current health care model, by shifting the focus from a ‘one-size-fits-all’ system to one that is patient-tailored, must provide clinicians with the right tools to treat the right patient with the right medicine at the right doses, and at the right time.

This two-stage systematic review and meta-analysis have been carried out following a protocol-registered review, which was prospectively updated [37]. Our intention was to prevent changes that can cause reporting biases [58] and to guarantee that our analysis was not a duplicate of previous ones [59,60]. Although the purposes at Stage 1 and 2 were different, together both stages were conceived with the intention to provide evidence on the clinical effect of vasopressin-receptor antagonists related to changes in serum sodium concentration. According to our two-stage systematic review design, evidence flowed from wide sources to in-focus sources, such as responses in the bias items of the Quality in Prognosis Studies (QuIPS) tool across the two steps conforming it, which follow the Wortman “mixed-criteria” approach [61]. In addition, the findings were put into context by using a multidisciplinary supervision mechanism, based on an expert advisory group in the topics addressed (fluid resuscitation and translational pharmacology).

This study has limitations. The comprehensiveness of our literature search could not be demonstrated mathematically, due to funnel plot asymmetry [44]. In addition, heterogeneity was large at each of the two stages [62]. However, variability between studies was expected. Sample characteristics (distinct molecules and dosages, patients with both hypervolemic and euvolemic hyponatremia at Stage 1, patients with two distinct diseases at Stage 2) can explain the statistical heterogeneity [63] that even increases in the tolvaptan subgroup (Stage 1) [64]. Finally, it should not be forgotten that only three RCTs were eligible at Stage 2, which may be a rationale for including observational studies. Observational evidence may provide moderate to high strength evidence in systematic reviews, but this is very rare, and it would mostly be indicated in assessing prognosis biomarkers [65].

## 5. Conclusions

In conclusion, our study shows that patients with worsening HF or with cirrhotic ascites, needing a treatment with vaptans, have better clinical outcomes when correction of hyponatremia (normalization or an increase in [Na^+^]_serum_ of at least 3–5 mEq/L) is achieved. Nevertheless, the evidence base needs to be enlarged to propose this treatment objective more formally as a new predictive or treatment stratification biomarker.

From the perspective of HTA, treatment stratification biomarkers must serve to improve outcomes related to the use of medicines. Nevertheless, despite advances, clinical qualification of potential candidates continues to be difficult [10]. Healthcare is changing, and there is an urgent need for targeted or more personalized therapies that, in our opinion, should be specially addressed to the most susceptible patient populations [66].

## Figures and Tables

**Figure 1:**
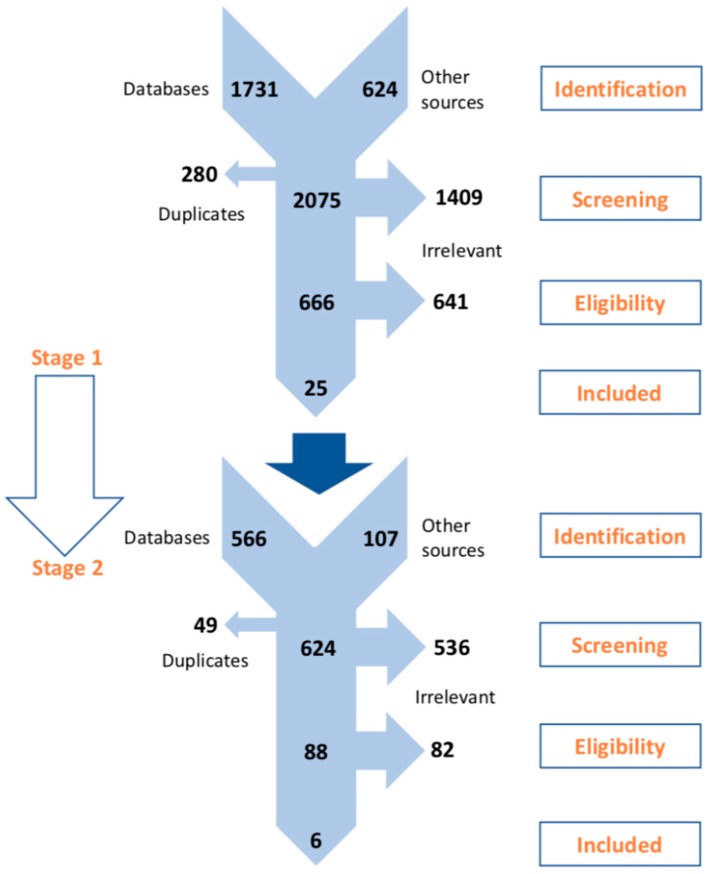
PRISMA flowchart presenting the selection process at the two stages. PRISMA, Preferred Reporting Items for Systematic Reviews and Meta-Analyses.

**Figure 2:**
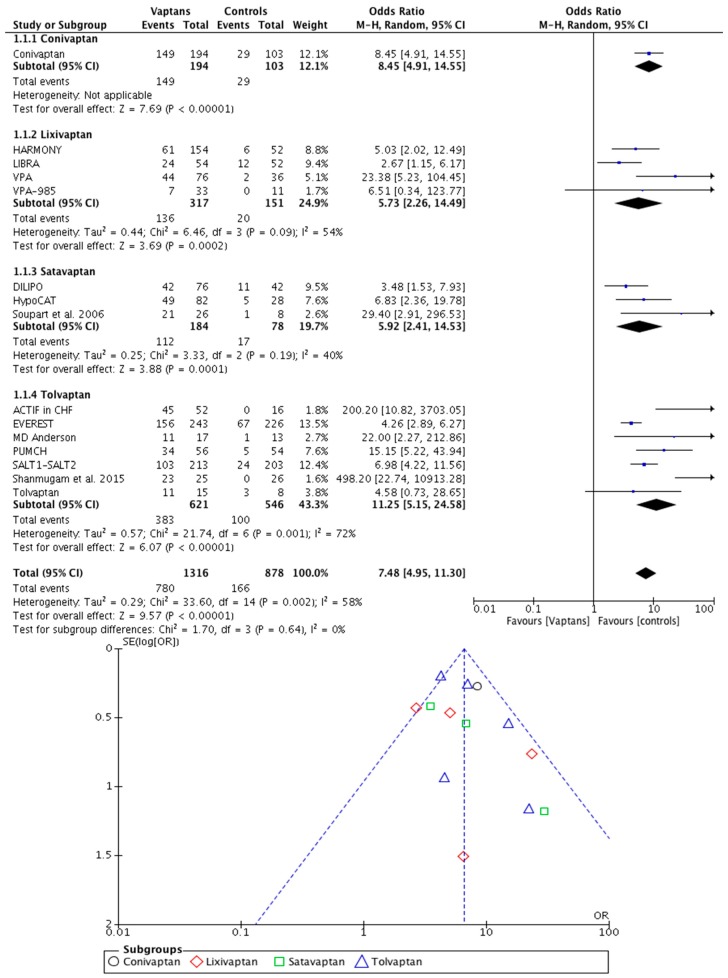
The effect of vaptans on serum sodium concentration. CI, confidence interval; M-H, Mantel–Haenszel test; SE, standard error.

**Figure 3:**
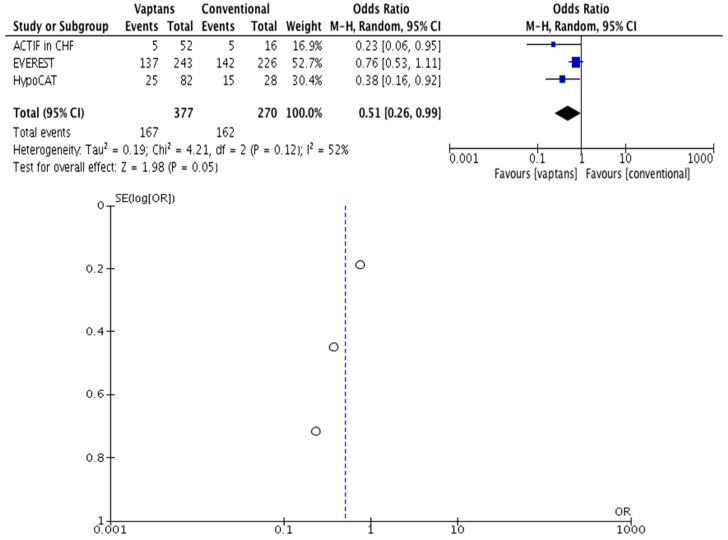
The effect of correction of hyponatremia on clinical outcomes in worsening HF and cirrhotic ascites.

**Table 1:** Specific stage review questions and study eligibility.

	Systematic Mapping (Stage 1)	In-Depth Meta-Analysis (Stage 2)
**Review Question**	Do vaptans have an influence on hyponatremia ^€^?	Is there an association between correction of hyponatremia ^$^ under vaptans and improvement of clinical outcomes in both worsening HF and cirrhotic ascites?
**Participants/** **Population**	Patients with hypervolemic/euvolemic hyponatremia of diverse causes	Patients with worsening HF or with cirrhotic ascites, having hyponatremia.
**Intervention(s)/** **Exposures(s)**	Vaptans	Correction of hyponatremia and improvement of the following clinical outcomes: rehospitalization and/or death in patients with worsening HF and ascites worsening in liver cirrhosis patients with ascites.
**Comparators**	Placebo/standard care	No correction of hyponatremia and no clinical improvement of worsening HF or cirrhotic ascites.

^€^ Hyponatremia: [Na^+^]_serum_ < 135 mEq/L. ^$^ Correction of hyponatremia: an increase in [Na^+^]_serum_ of at least 3–5 mEq/L from Days 2–14. Abbreviations: [Na^+^]_serum_, serum sodium concentration, HF, heart failure.

**Table 2:** Participants, interventions, comparators and outcomes in eligible studies.

Trials Details	Design	Follow-Up	Participants/Population Characteristics	Interventions (n)	Comparators (n)	Outcomes	Co-Interventions
**Conivaptan** Global [12,13,14,15]	RCT	7–9 days	Males (%)/≥65 year (%): 52.31/68.48. [Na^+^]_serum_ < 130 mEq/lt. Causes (%): SIADH (51.44), HF/COPD (32.30), cancer (11.11), postsurgical (4.47).	Conivaptan 40 mg/day (98) or 80 mg/day (96), IV/PO.	Placebo (103)	Day-5 [Na^+^]_serum_. Efficacy outcomes. ^£^	Fluid restriction to <2.0 L/24 h. Dietary and medication restrictions.
**HARMONY** NCT00876798 Global [16]	RCT	24 weeks	Males (%)/≥65 year (%): 49.70/51.42. [Na^+^]_serum_ < 135 mEq/lt Causes (%): SIADH (98.00), cancer (2.00).	Lixivaptan 25 mg plus dose titration (154).	Placebo (52)	Day-7 [Na^+^]_serum_.	Fluid restriction (investigator’s discretion).
**LIBRA** NCT00660959 Global [17]	RCT	30 days	Males (%)/≥65 year (%): 53.05/54.51. [Na^+^]_serum_ < 130 mEq/L Causes (%): SIADH (92.50), cancer (7.50).	Lixivaptan 50 mg plus dose titration (54).	Placebo (52)	Day-7 [Na^+^]_serum_.	Fluid restriction (investigator’s discretion).
**VPA** Europe [18,19]	RCT	7 days	Males (%): 76.79. [Na^+^]_serum_ < 130 mEq/lt Causes (%): liver cirrhosis (55.29), SIADH (29.53), HF (13.46).	Lixivaptan 100 mg/day (36) or 200 mg/day (40).	Placebo (36)	Day-7 [Na^+^]_serum_.	Fluid restriction to <1.0 L/24 h.
**VPA-985** Europe [20]	RCT	9 days	Males (%): 70.52. [Na^+^]_serum_ < 130 mEq/lt Causes (%): liver cirrhosis (75.00), HF (13.60), SIADH (11.40).	Lixivaptan 25 mg/day (12), 125 mg/day (11), or 250 mg/day (10)	Placebo (11)	Day-7 [Na^+^]_serum_.	Diuretics Fluid restriction to <1.5 L/24 h. Dietary restrictions.
**DILIPO**^#^ NCT00274326 Global [21]	RCT	48 weeks	Males (%)/≥65 year (%): 57.03/38.42. [Na^+^]_serum_ < 135 mEq/lt Causes (%): HF (76.44), SIADH (17.17), postsurgical (4.35).	Satavaptan 25 mg/day (35) or 50 mg/day (41).	Placebo (42)	Day-2 [Na^+^]_serum_. Efficacy outcomes. ^£^	Fluid restriction to <1.5 L/24 h.
**Soupart et al., 2006** Europe [22]	RCT	12 months	Males (%)/≥65 year (%): 57.03/38.41. [Na^+^]_serum_ < 135 mEq/lt Causes (%): SIADH (85.67), cancer (14.33).	Satavaptan 25 mg/day (14) or 50 mg/day (12).	Placebo (9)	Day-5 [Na^+^]_serum_. Efficacy outcomes. ^£^	Fluid restriction to <1.5 L/24 h.
**HypoCAT**^&^ NCT00501722 Europe [23]	RCT STR ^¥^	14 days	Males (%): 70.05. [Na^+^]_serum_ < 130 mEq/lt Cause: ascites in liver cirrhosis.	Satavaptan 5 mg/day (28), 12.5 mg/day (26) or 25 mg/day (28).	Placebo (28)	Day-5 [Na^+^]_serum_ in association to clinical outcomes at Day-30.	Fluid restriction to <1.5 L/24 h.
**SALT1–SALT2**^†^ NCT00072683 NCT00201994 Global [24,25]	RCT	37 days	Males (%): 58.33. [Na^+^]_serum_ < 135 mEq/lt Causes (%): SIADH (42.70), HF (30.75), liver cirrhosis (26.55).	Tolvaptan 15 mg/day (225)	Placebo (223)	Day-4 and Day-30 [Na^+^]_serum_. Efficacy outcomes. ^£^	Medication restrictions.
**ACTIF in CHF**^□^ Global [26,27]	RCT STR ^¥^	60 days	Males (%): 69.10. [Na^+^]_serum_ < 135 mEq/lt Cause: HF.	Tolvaptan 30 mg/day (15), 60 mg/day (22), or 90 mg/day (15).	Placebo (16)	Day-3 [Na^+^]_serum_ in association to clinical outcomes at Day-60.	HF therapy.
**EVEREST**^□^ NCT00071331 Global [28,29,30]	RCT STR ^¥^	60 days	Males (%): 76.48. [Na^+^]_serum_ < 135 mEq/lt Cause: HF.	Tolvaptan 30 mg/day (243)	Placebo (232)	Day-3 [Na^+^]_serum_ in association to clinical outcomes at Day-60.	HF therapy.
**Tolvaptan** USA [31]	RCT	65 days	Males (%): 57.00. [Na^+^]_serum_ < 135 mEq/lt Causes (%): HF (50.00), SIADH (36.00), liver cirrhosis (14.00).	Tolvaptan 10 mg/day plus dose titration (17).	Placebo (11)	Day-5 [Na^+^]_serum_. Efficacy outcomes. ^£^	Fluid restriction to <1.2 L/24 h.
**PUMCH**^‡^ NCT00664014 China [32,33,34]	RCT	7 days	Males (%): 51.11. [Na^+^]_serum_ < 135 mEq/lt Causes (%): HF (59.90), SIADH (40.10).	Tolvaptan 15 mg/day plus dose titration (56).	Placebo (54)	Day-4 and Day-7 [Na^+^]_serum_. Efficacy outcomes. ^£^	Fluid restriction (investigator’s discretion).
**MD Anderson Cancer Center** NCT01199198 USA [35]	RCT	14 days	Males (%): 53.57. [Na^+^]_serum_ < 130 mEq/lt Causes (%): cancer (89.00), SIADH (11.00).	Tolvaptan 15 mg/day plus dose titration (17).	Placebo (13)	Day-14 [Na^+^]_serum_.	DiureticsFluid restriction to <1.5 L/24 h.
**Shanmugam et al., 2015** CTRI/2013/05/003643 [36] India	RCT	30 days	Males (%): 70.70. [Na^+^]_serum_ < 135 mEq/lt Cause: HF.	Tolvaptan 15 mg/day (25)	Placebo (26)	Day-5 [Na^+^]_serum_.	None

^£^ Efficacy outcomes were those related to electrolyte free-water excretion. ^¥^ Post-randomization stratification of participants according to changes in [Na^+^]_serum_. ^□^ Data on hyponatremic participants are provided. ^#^ The DILutional hyPOnatremia (DILIPO) study. ^&^ Satavaptan dose-ranging study in Hyponatremic patients with Cirrhotic AsciTes (HypoCAT) study. ^†^ Study of Ascending Levels of Tolvaptan in Hyponatremia 1 and 2 (SALT1 and SALT2). ^‡^ Study carried out by the Peking Union Medical College Hospital (PUMCH). Abbreviations: [Na^+^]_serum_, serum sodium concentration; COPD, chronic obstructive pulmonary disease; HF, heart failure; IV, intravenous; PO, per oral; STR, stratified randomization; RCT, randomized controlled trial; SIADH, syndrome of inappropriate antidiuretic hormone secretion.

**Table 3:** Assessment of codependency when combining the treatment and the biomarker.

Information Requests	Comments
**Section 1 : Context**	
**Details about the Biomarker, the Test and the Medicine**	
1 (O) Current reimbursement arrangements.	Changes in [Na^+^]_serum_ would permit evaluation of treatment effect or response to vaptans in patients with worsening HF or cirrhotic ascites. Testing is widely available and affordable.
2 (T) Test sponsor.	Three methods (flame photometry, indirect and direct potentiometry) and many sponsors are currently available to measure sodium levels in serum.
3 (M) Medicine sponsor.	Otsuka: Samsca^®^ (tolvaptan).
4 (O) Biomarker.	Correction of hyponatremia: normalization or increase of [Na^+^]_serum_ of at least 3–5 mEq/L after 2–5 days of treatment with vaptans.
5 (T) Proposed test.	Determination of serum sodium.
6 (O) Medical condition or problem being managed.	Worsening HF and cirrhotic ascites.
7 (O) Clinical management pathways.	Decision-making in the management of patients with worsening HF or cirrhotic ascites under treatment with vaptans.
**Rationale for the Codependency**	
8 (O) Definition of the biomarker.	Treatment stratification biomarker.
9 (O) Biological rationale for targeting that biomarker(s).	Correction of hyponatremia could be associated with favorable clinical outcomes in patients with worsening HF and cirrhotic ascites.
10 (O) Other biomarker(s) to assess treatment effect of the medicine.	NA
11 (O) Prevalence of the condition being targeted in the population that is likely to receive the test.	The conditions are very prevalent.
**Proposed Impact of Codependent Technologies on Current Clinical Practice**	
12 (T) Consistency of the test results over time.	Clinical outcomes in both worsening HF and cirrhotic ascites improved under the effect of vaptans if correction of hyponatremia was achieved.
13 (T) Use of the proposed test with other treatments and/or for other purposes.	NA
14 (T) Use of the test in the clinical management pathway.	The test is most likely to be an additional test for managing patients.
15 (T) Provision of the test.	The test is in routine use worldwide.
16 (T) Specimen or sample collection.	Blood serum
17 (T) Use of the test for monitoring purposes (if relevant)	For identifying good and poor responders to vaptans.
18 (O) Availability of other tests for the biomarker.	None
**Section 2: Clinical Evaluation**	
**Direct Evidence Approach**	
**Section 2a: Evidence of Prognostic Effect of the Biomarker**	
19 (O) Prognostic effect of the biomarker.	Not assessed.
**Section 2d: Clinical Evaluation of the Codependent Technologies (Combined)**	
20 (O) Selection of the direct evidence.	Direct evidence, albeit of a lower level, is provided by retrospective biomarker-stratified RCTs.
21 (O) Quality of the direct evidence.	Adequate quality.

Item numbers are tagged with (T), (M) or (O), which indicate whether the item number is relevant to the test, the medicine or overlaps both. Abbreviations: [Na^+^]_serum_, serum sodium concentration; HF, heart failure; NA, not available; RCT, randomized controlled trial.

## Supplementary Materials

The following are available online at http://www.mdpi.com/2077-0383/7/9/262/s1: Table S1: Assessment of risk of bias in eligible studies.
