# Supplementary material for: Correction of Hyponatremia May Be a Treatment Stratification Biomarker: A Two-Stage Systematic Review and Meta-Analysis
Source: J Clin Med. 2018 Sep 7;7(9):262. doi: 10.3390/jcm7090262 (PMC6162844; doi:10.3390/jcm7090262)
Supplement: Supplementary file 1 [file jcm-07-00262-s001.pdf]

Supplementary Table S1. Assessment of risk of bias in eligible studies.

Trials	Random sequence generation	Allocation concealment	Blinding of participants and personnel	Blinding of outcome assessment	Incomplete outcome data	Selective reporting	Other bias
**Conivaptan**	L	L	U	U	U	U	U
**HARMONY**	L	L	U	U	U	L	U
**LIBRA**	L	L	U	U	U	L	U
**VPA**	L	L	U	U	L	U	U
**VPA-985**	L	L	U	U	L	U	U
**DILIPO**	L	L	U	U	L	L	U
**Soupart et al., 2006**	L	L	U	U	L	U	U
**HypoCAT**	L	L	U	U	L	L	U

**SALT1-SALT2**	L	L	U	L	U	L	U
**ACTIF in CHF**	L	L	U	U	U	U	U
**EVEREST**	L	L	U	U	L	L	U
**Tolvaptan**	L	L	U	U	U	U	U
**PUMCH**	L	L	U	U	U	U	U
**MD Anderson Cancer Center**	L	L	U	U	U	L	U
**Shanmugam et al., 2015**	L	L	U	U	L	L	L

Judgement for each of the risk of bias domains is presented as L, U or H, indicating, respectively, low, unclear or high risk of bias.
